# Halogen Bond-Involving Self-Assembly of Iodonium Carboxylates: Adding a Dimension to Supramolecular Architecture

**DOI:** 10.3390/ijms241914642

**Published:** 2023-09-27

**Authors:** Amirbek D. Radzhabov, Alyona I. Ledneva, Natalia S. Soldatova, Irina I. Fedorova, Daniil M. Ivanov, Alexey A. Ivanov, Mekhman S. Yusubov, Vadim Yu. Kukushkin, Pavel S. Postnikov

**Affiliations:** 1 Research School of Chemistry and Applied Biomedical Sciences, Tomsk Polytechnic University, Tomsk 634050, Russiasoldatovans@tpu.ru (N.S.S.); d.m.ivanov@spbu.ru (D.M.I.); yusubov@tpu.ru (M.S.Y.); 2 Institute of Chemistry, Saint Petersburg State University, Saint Petersburg 199034, Russiav.kukushkin@spbu.ru (V.Y.K.); 3 Department of Mathematics and Mechanics, Saint Petersburg State University, Saint Petersburg 199034, Russia; 4 Institute of Chemistry and Pharmaceutical Technologies, Altai State University, Barnaul 656049, Russia; 5 Department of Solid State Engineering, Institute of Chemical Technology, 16628 Prague, Czech Republic

**Keywords:** halogen bond, diaryliodonium salt, carboxylate, supramolecular assembly, DFT calculations

## Abstract

We designed 0D, 1D, and 2D supramolecular assemblies made of diaryliodonium salts (functioning as double σ-hole donors) and carboxylates (as σ-hole acceptors). The association was based on two charge-supported halogen bonds (XB), which occurred between I^III^ sites of the iodonium cations and the carboxylate anions. The sequential introduction of the carboxylic groups in the aryl ring of the benzoic acid added a dimension to the 0D supramolecular organization of the benzoate, which furnished 1D-chained and 2D-layered structures when terephthalate and trimesate anions, correspondingly, were applied as XB acceptors. The structure-directing XB were studied using DFT calculations under periodic boundary conditions and were followed by the one-electron-potential analysis and the Bader atoms-in-molecules topological analysis of electron density. These theoretical methods confirmed the existence of the XB and verified the philicities of the interaction partners in the designed solid-state structures.

## 1. Introduction

Halogen bonding (abbreviated as XB) [1,2], as a part of the spectrum of “unorthodox” [3] noncovalent interactions, is a subject of growing interest in crystal engineering [4,5,6], biomedical science [7,8,9,10], ion and molecular recognition [11,12,13,14], noncovalent catalysis [15,16,17,18], and many other fields [19]. The particular interest in XB lies in the area of crystal engineering and the high directionality of XB is a main factor for the rational design of the targeted supramolecular architectures.

Modern XB-based crystal engineering mainly utilizes monovalent halogen organic compounds, exhibiting one σ-hole per one halogen(I) site. In the vast majority of instances, these atoms form two-center XB. For the crystal design of higher-dimensional arrays, polyhalogenated XB donors—in which every halogen site provides one σ-hole for the appropriate XB—should be applied. 

A suitable alternative to the polyhalogenated compounds is a hypervalent halogen species such as a diaryliodonium salt-bearing I^III^ site as a double σ-hole donor [20,21,22]. Diaryliodonium salts have already been used for the control of solid-state reactions [23], the stabilization of copper(I) complexes [24], the design of extended supramolecular arrays [25,26,27], XB-involving catalysis [17,18,28,29,30], and for the preparation of iodonium-based porous materials [31].

**Figure 1:**
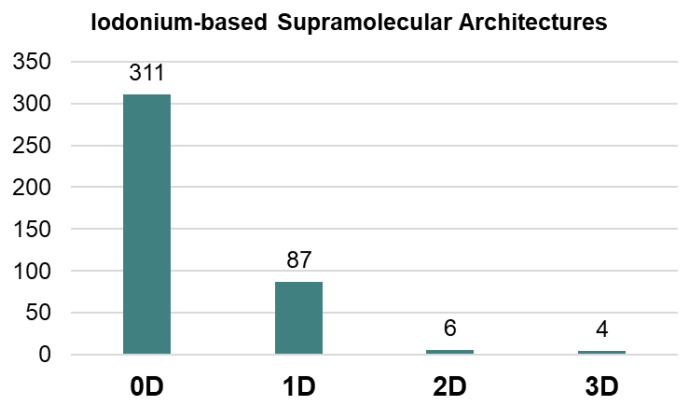
Supramolecular arrays of different dimension from CSD.

Our processing of the Cambridge Structural Database (CSD) showed that highly dimensional supramolecular architectures, namely 2D layers and 3D frameworks, based on iodonium species are still quite rare (<3%; Figure 1). The most common motifs in the supramolecular organization of iodonium species are 0D clusters or 1D-chained arrays (scattered examples) (Figure 1). The utilization of iodonium cations as tectons for the rational construction of highly dimensional supramolecular architectures is poorly studied and, in fact, it is limited by our findings in the design of halogen-bonded 1D chains of solid iodonium disulfonates [25]. Notably, in the case of iodonium sulfonates, we also obtained a few 2D-layered structures from the uncontrolled crystal growth.

Inspired by our success in the rational design of 1D-chained architectures from iodonium disulfonates, we extended this approach to other salts, namely iodonium carboxylates—benzoates, terephthalates, and trimesates. These derivatives of benzoic acid are commercially available and they have been repeatedly employed in the syntheses and design of metal–organic frameworks [32]. In comparison with iodonium sulfonates, the structures of the corresponding carboxylates are poorly studied and available examples are limited only to iodonium acetates; trifluoroacetates (~20 structures); and to one structure of an iodonium benzoate [33].

**Figure 2:**
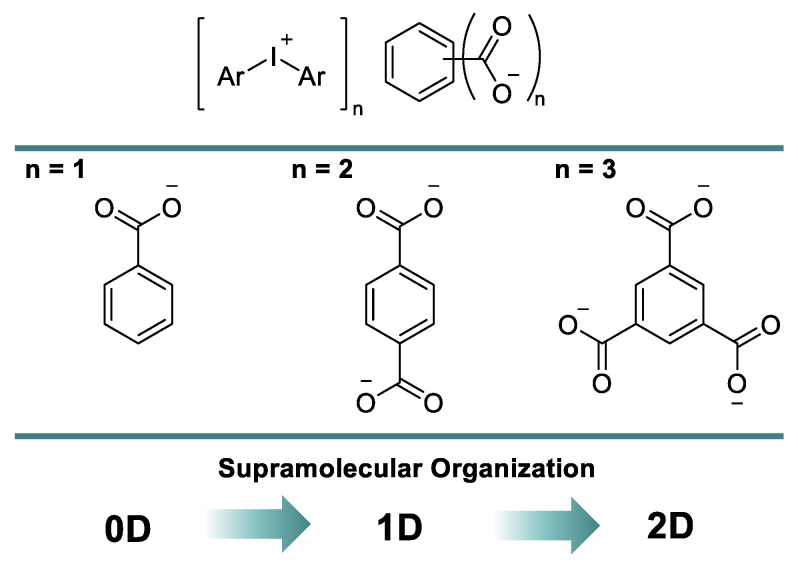
Adding a dimension to the supramolecular organization of iodonium carboxylates.

In this report, we assumed that the sequential introduction of carboxylic groups in the aryl ring of benzoic acid could increase the dimension of corresponding XB-based supramolecular assemblies. In this way, one could design different supramolecular architectures using a variation of a carboxylate anion of iodonium salts and obtain 0D clusters for benzoates, 1D chains for terephthalates, and 2D layers for trimesates (Figure 2). All of our findings are detailed in the following sections.

## 2. Results and Discussion

### 2.1. Synthesis and Crystal Growth 

**Figure 3:**
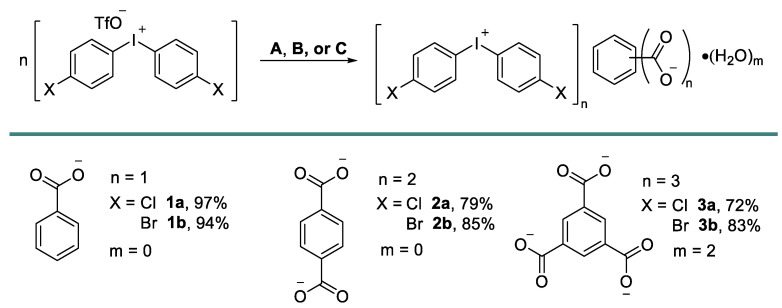
Preparation of **1**–**3**. Conditions **A**: iodonium triflate (1 equiv.), potassium benzoate (3 equiv.), MeOH/H_2_O; Conditions **B**: iodonium triflate (2.2 equiv.), (Bu_4_N^+^)_2_C_6_H_4_(COO^−^)_2_ (1 equiv.), MeOH/H_2_O; Conditions **C**: iodonium triflate (3.3 equiv.), (Bu_4_N^+^)_3_C_6_H_3_(COO^−^)_3_ (1 equiv.), MeCN/H_2_O.

Iodonium carboxylates [33] were prepared in high isolated yields via the anion metathesis of potassium, or the Bu_4_N salts of corresponding carboxylates (TBA carboxylates), and iodonium triflates (Figure 3). Notably, the deviation from the reported benzoate load or concentration variations led to the contamination of the resulting product by triflates. The role of the solvent was also important as, for example, the change of solvent to neat MeCN or neat MeOH did not lead to the precipitation of the pure products. 

Crystals of **1** were grown on the slow evaporation of its MeOH solution at room temperature in air. Crystals of **2** and **3** were prepared via the co-crystallization of iodonium triflate with TBA carboxylates from aqueous MeCN, also at room temperature in air.

### 2.2. General Consideration of the XRD Structures

**Figure 4:**
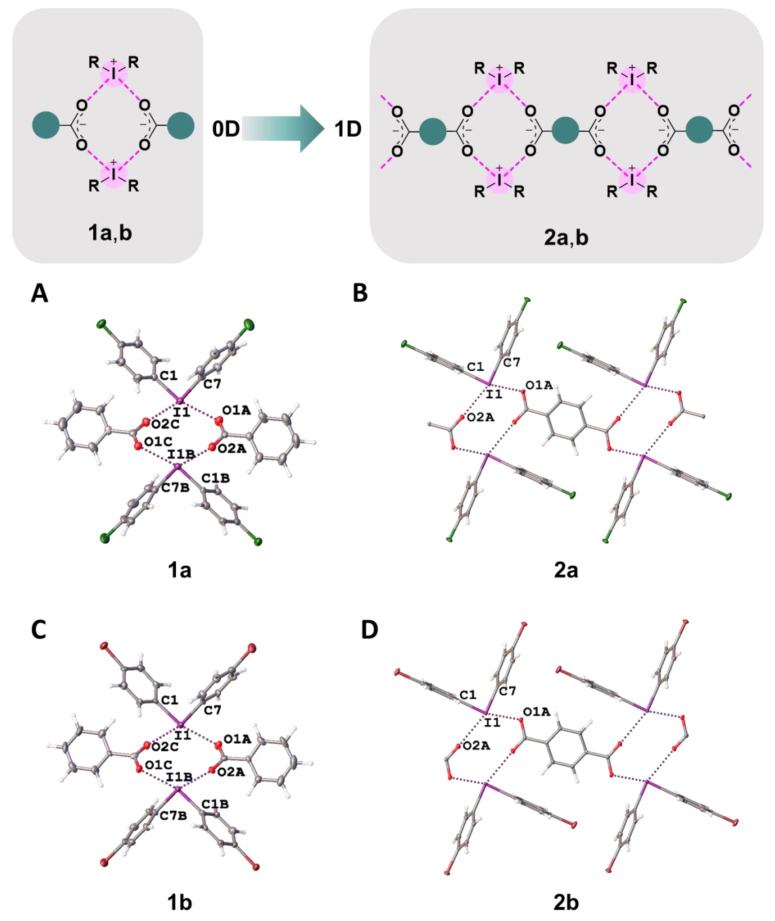
Graphical representation of **1a**,**b** and **2a**,**b** (top). Fragments of the crystal structures of **1a** (**A**), **2a** (**B**), **1b** (**C**), and **2b** (**D**) (middle and bottom).

**Figure 5:**
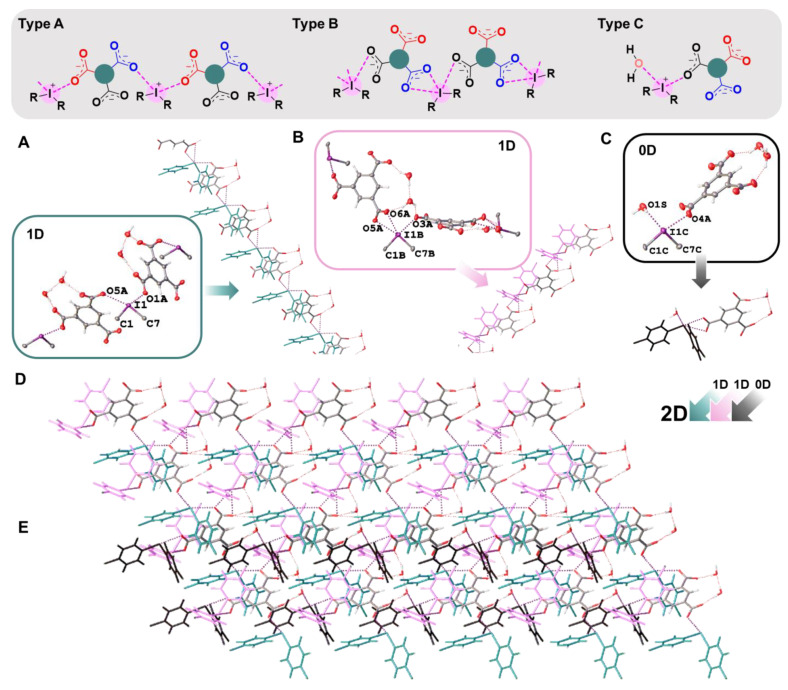
Graphical presentation of major motifs in **3a**,**b** (top). Fragments of the crystal structures **3a** and **3b**. (**A**,**B**): 1D chain from the assembly of the iodonium cations (A—Type A; B—Type B) with trimesate anion; (**C**): 0D structure from the assembly of iodonium cations (Type C) with trimesate anion; (**D**): 2D layer from the assembly of iodonium cations (Types A and B) with trimesate anion; (**E**): 2D layer from the assembly of iodonium cations (Types A, B, and C) with trimesate anion (middle and bottom).

In the crystal structures of **1a**,**b**–**3a**,**b**, the hypervalent I-atom forms two I∙∙∙O XBs with either the O-atoms of two carboxylic groups (Figure 4 and Figure 5A,B), or with the O-atom of a carboxylic group and the O-atom of a water molecule (Figure 5C). All these I∙∙∙O interactions fulfill the IUPAC geometrical criteria [1] for the identification of XB (*d*(I∙∙∙O) = 2.5–3.0 Å vs. ∑_vdW_ O + I = 3.5 Å [34]; ∠C–I∙∙∙X = 163–175°; Table 1). The only deviation from the general trend is the structures of **3a**,**b** (Figure 5B), in which the I^III^ site is involved in the bifurcated C7B–I1B∙∙∙O5A(O6A) XB of the type μ-I∙∙∙(O,O) (for more information on bifurcated XBs, see refs. [35,36,37,38]). The bifurcation is realized for iodonium cations of Type B (Figure 5B; hereinafter crystallographically independent iodonium cations in the same structure are defined as Type A, B, or C; Table 1) and the occurrence of the bifurcated XB was confirmed using appropriate DFT calculations (Section 2.4).

In the structures of **1a**,**b**–**3a**,**b**, the mean value of normalized contacts (Nc 0.78) for the I∙∙∙O XB, involving the carboxylic group which acted as an XB acceptor, agreed well with the Nc mean value (Nc 0.79) for other iodonium carboxylates from CSD. Further inspection of CSD and the comparison of I∙∙∙O XBs, including the carboxylic (this work) or a sulfonate group (accessed in CSD), revealed that Nc values for iodonium carboxylates (our data 0.78; CSD data: mean value 0.79) are lower than those for iodonium sulfonates (mean value 0.82). This comparison indirectly indicates that the carboxylate systems provide stronger XBs, probably due to a more localized negative charge on the carboxylate function (bearing two electronegative O-atoms), rather than that on the sulfonate group (featuring three O-atoms).

All pairs of structures (namely, **1a** and **1b**, **2a** and **2b**, and **3a** and **3b**) of the salts bearing *p*-Cl (for **1a**–**3a**) and *p*-Br substituents (**1b**–**3b**) in the arene rings provided examples of the isostructural exchange [39,40,41,42] (Figure S1). The counterions did not affect this exchange and, furthermore, crystal packings were the same for the *p*-Cl and *p*-Br substituents. Previously, we reported a relevant isostructural exchange in symmetrical [43] and unsymmetrical [23] iodonium salts bearing *p*-Cl and *p*-Br substituents in arenes of iodonium cations.

Notably, in the structures of **1a**,**b** and **3a**,**b**, the halogens of the arene rings formed additional X∙∙∙X (X = Cl or Br). These XBs occurred between a σ-hole of one halogen and an electron belt of another halogen atom. However, these interactions were characterized by rather large Nc values (~1.00), indicating that they were very weak (Table S1). The angles ∠C–X∙∙∙X (163–177°) were close to 180°, and this, in combination with the results of the DFT calculations (Section 2.4), allowed the attribution of these interactions to XBs, according to the IUPAC classification [44]. In comparison with the C–X∙∙∙X XBs, stronger C–X∙∙∙O XBs were observed in the structures of **3a**,**b**, in which the Nc was noticeably lower than 1.00 (X = Cl, Nc = 0.94; X = Br, Nc = 0.91), although the angles ∠C–X∙∙∙O (~155°; Table S1) deviated from linearity.

### 2.3. XRD Structures: Supramolecular Assembly 

In **1a**,**b** and **2a**,**b**, two cationic and two anionic species assembled into heterotetrameric motifs via four I∙∙∙O XBs (Figure 4). Similar heterotetrameric motifs were found in a large number of iodonium salts, in particular, in the structure of iodonium benzoate (CSD refcode: TUDWEX) [33]. In the cases of benzoates **1a**,**b**, the crystal structures exhibited 0D organization, while the addition of one carboxylic group in terephthalates **2a**,**b** increased the dimensionality providing assembly into 1D chains by linking the heterotetrameric motifs to the phenylene bridges, –C_6_H_4_– (Figure 4). 

We earlier reported a relevant self-assembly of iodonium disulfonates, where heterotetrameric motifs were linked by naphthalene bridges [25]. Apart from 1D chains, the studied iodonium disulfonates formed 2D-layered structures [25]. However, occurrence of the 2D systems happened occasionally, depending on cation, anion, and crystallization conditions. 

We assumed that for the triple-charged anion (namely, trimesate anion), the occurrence of 2D layers was more favorable due to the branching of the supramolecular assembly by a larger number of XB-accepting sites in the same functionality. According to our expectations, the replacement of doubly-charged terephthalates in **2a**,**b,** to triply-charged trimesates in **3a**,**b,** led to the addition of a dimension and accomplished the 2D-layered architecture. The structures of **3a**,**b** included one trimesate anion, three crystallographically independent iodonium cations (Types A–C), and two water molecules; the latter were linked to a trimesate anion by a hydrogen bond (namely, O2A···*H–O1S–H*···O2S and O6A···*H–O2S–H*···O3A, Figure S2).

In general, the analysis of the crystal structures of **3a**,**b** revealed five different XBs with trimesate anions, namely four two-center and one three-center bifurcated XBs. Thus, 2D layers in **3a**,**b**—depending on the identity of the iodonium cation (Figure 5A–C)—exhibited three basic motifs. Type A and B cations formed 1D chains with trimesate anions (Figure 5A,B), whilst Type C iodonium cation formed a 0D cluster, including one trimesate anion and one H_2_O (Figure 5C). Both 1D-chained motifs displayed a similar architecture, where one trimesate anion interacted with two anions of one type (Type A: I1···O1A and I1···O5A XBs; Type B: I1B···O3A and I1B···O5A(O6A) XBs, Figure 5A,B). A combination of 1D chains (Types A and B) led to an XB net-like organization (Figure 5D), in which trimesate anions functioned as nodes. Each trimesate anion additionally interacted with Type C cations, so the 0D clusters motif was woven into XB net-like 2D layers (Figure 5E).

### 2.4. Theoretical Calculations

To closely interrogate the observed XB contacts, we performed appropriate DFT calculations, which were based on the experimentally determined XRD coordinates and performed under the periodic boundary conditions (crystal models, PBE [45]-D3 [46,47] level of theory, and the DZVP-MOLOPT-SR-GTH/SZV-MOLOPT-SR-GTH [48] bases within the Gaussian/plane wave (GPW) [49] methodology in CP2K). The DZVP-MOLOPT-SR-GTH basis set was used for all atoms in the structures of **2a** and **2b**. In view of software limitations for the structures exhibiting large unit cell volumes (>2000 Å^3^), the same approach for **1a**, **1b**, **3a**, and **3b** was not able to be performed and, hence, the calculations were conducted using the DZVP-MOLOPT-SR-GTH basis set for halogen atoms; O-atoms; and for the C-atom which is covalently bound to halogen or O-atoms; and also for H-atoms covalently bound to oxygen. The SZV-MOLOPT-SR-GTH basis set was used for the remaining H- and C-atoms.

The existence and noncovalent nature of the studied interactions was confirmed by the topological analysis of electron density within the Bader quantum theory of atoms in molecules (QTAIM analysis) [50,51,52,53]. Bond critical points (3, −1) (BCPs) between the iodonium I-atoms and the carboxylate O-atoms (including the bifurcate I···OCO interactions in **3a** and **3b**) were found, and they are gathered in Table 2. In addition, BCPs were detected between the Cl- (or Br) atoms in the structures of **1a**, **1b**, **3a**, and **3b**; between the Cl- (Br) atoms and the π-systems of the aromatic rings in **2a** and **2b**; and between the Cl- (Br) atoms and the carboxylate O-atoms in **3a** and **3b** (Table S2). Finally, BCPs between the H-atoms of H_2_O molecules and the O atoms of carboxylate were also identified.

The obtained BCP values of sign(*λ*_2_) *ρ*(**r**) were negative and small, and their considerations point to the attractive and noncovalent nature of the interactions under study [54]. Furthermore, these interactions can also be classified as noncovalent because of their close to zero positive energy density values (0.0002−0.0023 Hartree/bohr^3^); the balance of the Lagrangian kinetic energy *G*(**r**); and the potential energy density *V*(**r**) (−*G*(**r**)/*V*(**r**) > 1) at the corresponding BCPs [53]. In some cases, when *d*(I···O) < 3 Å or *d*(H···O) < 1.85 Å, the energy density values were negative, and this indicated a certain degree of covalency in the occurrence of these contacts.

**Figure 6:**
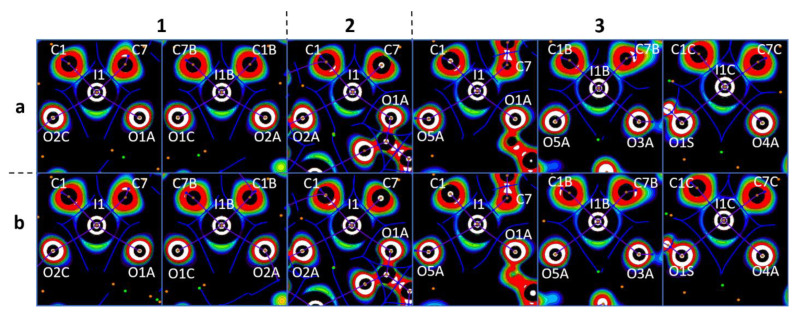
Visualization of the OEP projections through the O···I···O planes for the crystal models (numbers above the figure represent the anion structures and letters means the cation structures). Contour lines are drawn from −0.25 to 0.25 OEP value with 0.05 step and with additional −0.60 contour line; the color range is white (<−0.60), from red (−0.25) to purple (0.25), and black (>0.25). QTAIM *ρ*(**r**) topological pale brown nuclear (3, −3), blue bond (3, −1), orange ring (3, +1), and green cell (3, +3) critical points are drawn with purple bond paths and blue interbasin paths.

To confirm the philicities [55,56,57] (the property of atom(s) to function as electron donor(s) (nucleophile(s)) or electron acceptor(s) (electrophile(s)) of the coformers, we computed one-electron-potential (OEP) [58,59] projections with assigned critical points and bond paths from *ρ*(**r**) QTAIM analysis (Figure 6). The OEP-based approach has previously been used [60,61,62] for the visualization of shared and lone electron pairs. In particular, this method has been applied to various diaryliodonium systems and many other relevant systems [25]. The OEP approach is more useful than the electron localization function (ELF) [63,64,65] method considering that the former does not directly depend on the wave function. Consequently, one can calculate OEP in any area using the electron density function (EDF) for core electrons [66]. 

**Figure 7:**
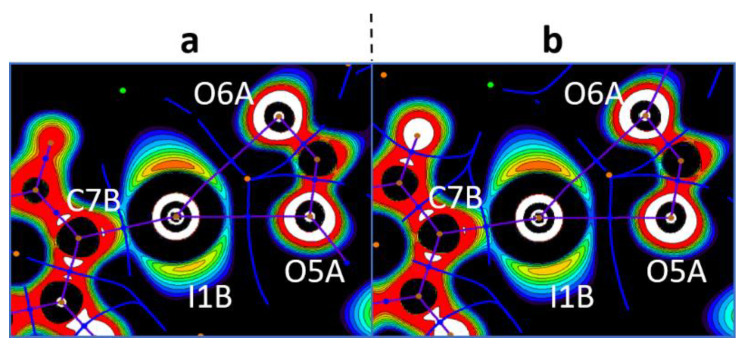
Visualization of the OEP projections through the O···I···O planes for the bifurcate I···OCO interactions (the crystal models **3a** (a) and **3b** (b)). Contour lines are drawn from −0.25 to 0.25 OEP value with 0.05 step and with additional −0.60 contour line; the color range is white (<−0.60), from red (−0.25) to purple (0.25), and black (>0.25). QTAIM *ρ*(**r**) topological pale brown nuclear (3, −3), blue bond (3, −1), orange ring (3, +1), and green cell (3, +3) critical points are drawn with purple bond paths and blue interbasin paths.

In all cases, the I···O bond paths passed between the I−C shared and iodine lone pair areas with positive OEP, namely through iodine σ-holes, and through the lone pair areas of the carboxylate O-atoms. This observation allowed the accurate determination of the philicities of the I- and O-atoms in the studied XBs, particularly the electrophilicity of the iodonium centers and the nucleophilicity of the carboxylate O-sites. The same pattern detected in the monofurcate was also detected for the bifurcate I···OCO interactions. In the latter case, bond paths were located between the lone pair areas around the iodonium I-atoms. This observation confirmed their electrophilicity toward the carboxylate O-atoms (Figure 7). Likewise, the analysis of the OEP projections verified the electrophilicity of the Cl- (Br) atoms; the nucleophilicity of the Cl- (Br) atoms; the C-atoms of the aromatic rings; and the O-atoms of carboxylate in X···X, X···C, and X···O (X = Cl, Br) interactions.

To summarize the computational results, we confirmed the occurrence of the I···O XBs and the Cl···Cl (Br···Br), Cl···C (Br···C) and Cl···O (Br···O) XBs, proved their noncovalent nature (albeit with a small covalent contribution), and determined the philicities of the coformers in the solid supramolecular assemblies.

## 3. Materials and Methods

### 3.1. General Information

All reagents and solvents were obtained from commercial sources and used without further purification. Iodonium salts were obtained using the previously reported procedure [1]. Melting points were measured on a BUCHI M-560 apparatus (BUCHI Labortechnik AG, Flawil, St. Gallen, Switzerland) in capillaries and were not corrected. The NMR spectra were recorded on Bruker Avance III HD (400 MHz) (Bruker Corp., Billerica, MA, USA). The ^1^H NMR spectra were recorded at 400 MHz and the ^13^C NMR spectra were recorded at 100 MHz. Chemical shifts were reported in parts per million (ppm). The ^1^H and ^13^C chemical shifts were referenced relative to the residual solvent signal. High-resolution mass spectra (HRMS) were recorded using electrospray ionization (ESI) methods on a Bruker micrOTOF spectrometer (Bruker Corp., Billerica, MA, USA) equipped with an ESI source. Elemental CHNS analysis was obtained on an elemental analyzer Thermo Flash EA 2000 (Thermo Fisher Scientific, Rockford, IL, USA), and sulfanilamide was used as a standard. Drying of the samples for elemental analysis was carried out at 80 °C to constant weight in a dry argon atmosphere using combined TG-DSC analysis on an SDT Q600 thermal analyzer (TA Instruments New Castle, DE, USA). 

### 3.2. X-ray Structure Determinations

X-ray diffraction data were collected at 100 K on a XtaLAB Synergy (Rigaku Oxford Diffraction, Oxford, UK), single-source at home/near, HyPix diffractometer using Cu Kα (λ = 1.54184 Å; **3a**,**b**) and a Tongda TD-5000 diffractometer (Dandong Tongda Science and Technology, Dangdong, China) using Mo Kα (λ = 0.71073; **1a**,**b**; **2a**,**b**). The structures were solved with the ShelXT (Shelx, Göttingen, Germany) [67] structure solution program using Intrinsic Phasing and refined with the ShelXL (Shelx, Göttingen, Germany) [68] refinement package incorporated in the OLEX2 program package (OlexSys Ltd., Durham, UK) [69] using Least Squares minimization. The XRD data and structural refinement parameters are summarized in Table S3. The hydrogen atoms in all structures were placed in ideally calculated positions according to neutron diffraction statistical data [70] and were refined as colliding atoms with parameters of relative isotropic displacement. Supplementary crystallographic data have been deposited at Cambridge Crystallographic Data Centre (CCDC 2291471–2291473, 2291475–2291477) and can be obtained free of charge via www.ccdc.cam.ac.uk/data request/cif (accessed on 30 August 2023). 

### 3.3. Computational Details 

Single-point DFT calculations under periodic boundary conditions were conducted using the mixed Gaussian/plane-wave (GPW) [49] basis set with 350 plane-wave; 50 Ry relative plane-wave cutoffs for the auxiliary grid; and the PBE [45]-D3 [46,47] level of theory for all studied crystals (1 × 1 × 1 cells) using the CP2K-8.1 program [71,72,73,74,75,76,77]. The PBE-D3 level of theory was previously applied for most of the CP2K calculations performed under 3D periodic boundary conditions [78,79,80,81,82,83,84,85,86,87]. In the structures of **2a** and **2b**, the DZVP-MOLOPT-SR-GTH basis set was applied for all atoms. However, to achieve 1.0 × 10^−6^ Hartree convergence for the self-consistent-field cycle in the Γ-point approximation, for the structures of **1a**, **1b**, **3a**, and **3b,** a combination of the DZVP-MOLOPT-SR-GTH and the SZV-MOLOPT-SR-GTH basis sets was applied. A similar methodology has previously been used for the studies of various halogen-bonded systems [88,89,90]. In some cases, the starting fractional coordinates were shifted along one (for **2b**) or two (for **2a**) translation vectors by 0.5 to move the heterotetrameric fragments (consisting of two anions and two cations) to the center of the cell. One-electron-potential (OEP) [58,59] analysis and Bader atoms in molecules topological analysis of electron density (QTAIM) [50,51,52,53] were performed and visualized in Multiwfn 3.8 [91]. The pseudopotential core areas were modeled by the inner code of Multiwfn 3.8 [66] for the OEP and QTAIM analyses.

### 3.4. Synthetic Procedures

#### 3.4.1. Preparation of Diaryliodonium Benzoates **1**

A solution of a diaryliodonium trifluoromethanesulfonate [92] (1 mmol) in methanol/water mixture (1 mL) was added dropwise to a solution of potassium benzoate (3 mmol, 481 mg) in water (5 mL) at RT. The reaction mixture was stirred for 30 min and the precipitate formed was filtered off and washed with water (3 × 5 mL). The prepared diaryliodonium benzoates **1** were dried under reduced pressure.

#### 3.4.2. Preparation of Diaryliodonium Terephtalates **2**

To a solution of a diaryliodonium trifluoromethanesulfonate [92] (2.2 mmol) in methanol/water mixture (5 mL, 1:1), the solution of tetrabutylammonium terephthalate (1 mmol, 649 mg) in methanol (1 mL) was added dropwise at RT. The reaction mixture was stirred for 30 min and the precipitate formed was filtered off and washed with water (3 × 5 mL). The prepared diaryliodonium terephthalates **2** were dried under reduced pressure.

#### 3.4.3. Preparation of Diaryliodonium Trimesates **3**

To a solution of tetrabutylammonium trimesate (1 mmol, 934 mg) in water/acetonitrile mixture (5 mL, 1:1), a solution of diaryliodonium trifluoromethanesulfonate [92] (3.3 mmol) in acetonitrile (5 mL) was added dropwise at RT. The reaction mixture was stirred for 30 min and the precipitate formed was filtered off and washed with water (3 × 5 mL). The prepared diaryliodonium trimesates **3** were dried under reduced pressure.

## 4. Conclusions

We utilized the iodonium carboxylates for the design of halogen-bonded supramolecular assemblies (0D, 1D, and 2D). Iodonium cations acted as double σ-hole XB donors, while the carboxylate anions functioned as efficient XB acceptors. The increase in the number of carboxylic groups led to the addition of a dimension to the supramolecular assemblies. Thus, the association of iodonium benzoates furnished a 0D cluster, whilst the use of the terephthalate species and the trimesate species furnished 1D-chained or 2D-layered structures, correspondingly. To model the solid-state electron wave function, DFT calculations under periodic boundary conditions were performed. A topological analysis of the electron density revealed the bond critical points for interionic XBs and, in the cases of **3a** and **3b**, for bifurcated I···(OCO) XBs. The projections of one-electron potential, which verified the electron pair positions, confirmed the electrophilicity of the XB donors.

## Figures and Tables

**Table 1:** Geometrical parameters of XBs in the structures of **1a**,**b**–**3a**,**b**.

Cation Type *^a^*	XB	*d*(C–I∙∙∙X)	∠(C–I∙∙∙X)	Nc *^b^*	*d*(C–I∙∙∙X)	∠(C–I∙∙∙X)	Nc *^b^*
		1a			1b		
A	C1–I1∙∙∙O1A	2.622(3)	164.04(12)	0.75	2.628(4)	164.19(19)	0.75
	C7–I1∙∙∙O2C	2.661(3)	164.55(13)	0.76	2.664(4)	164.39(19)	0.76
B	C1B–I1B∙∙∙O1C	2.663(3)	163.96(12)	0.76	2.669(4)	163.39(19)	0.76
	C7B–I1B∙∙∙O2A	2.650(3)	164.87(12)	0.76	2.647(4)	164.17(19)	0.76
		**2a**			**2b**		
	C1–I1∙∙∙O1A	2.479(2)	165.00(9)	0.71	2.480(2)	163.95(9)	0.71
	C7–I1∙∙∙O2A	2.9964(18)	164.99(9)	0.86	2.9612(18)	163.98(9)	0.85
		**3a**			**3b**		
A	C1–I1∙∙∙O1A	2.479(6)	168.4(3)	0.71	2.474(4)	167.7(2)	0.71
	C7–I1∙∙∙O5A	2.957(6)	164.2(2)	0.84	2.956(5)	164.00(17)	0.84
B	C1B–I1B∙∙∙O3A	2.692(6)	174.5(3)	0.77	2.697(5)	170.3(2)	0.77
	C7B–I1B∙∙∙O5A	2.833(6)	166.8(2)	0.81	2.766(5)	169.5(2)	0.79
	C7B–I1B∙∙∙O6A	3.037(6)	148.1(2)	0.87	3.097(4)	144.8(2)	0.88
C	C1C–I1C∙∙∙O4A	2.630(6)	171.2(3)	0.75	2.640(5)	171.03(19)	0.75
	C7C–I1C∙∙∙O1S	2.828(8)	172.5(3)	0.81	2.771(5)	173.3(2)	0.79

*^a^* Applicable if the crystal structure exhibits several types of crystallographically independent iodonium cations; *^b^* the normalized contact (Nc) is defined as the ratio between the separation observed in the crystal and the sum of Bondi vdW radii of interacting atoms: Nc = d/Σ_vdW_; Σ_vdW_(I + O) = 3.50 Å.

**Table 2:** Parameters in (3, −1) bond critical points (the electron density with sign of *λ*_2_ sign(*λ*_2_)*ρ*(**r**) in *e*/bohr^3^, Laplacian of electron density ∇^2^*ρ*(**r**) in *e*/bohr^5^, the local electronic energy density *H*_b_, local electronic potential energy density *V*(**r**), local electronic kinetic energy density *G*(**r**) in Hartree/bohr^3^) corresponding to the I∙∙∙O XBs in crystal models of all structures.

Structure	Contact	*l*	Sign(*λ*_2_)*ρ*(r)	∇^2^*ρ*(r)	*V*(r)	*G*(r)	*H*_b_
**1a**	I1···O1A I1···O2C I1B···O2A I1B···O1C	2.622 2.661 2.650 2.663	−0.0349 −0.0321 −0.0330 −0.0325	0.1008 0.0948 0.0960 0.0936	−0.0260 −0.0234 −0.0241 −0.0234	0.0247 0.0229 0.0233 0.0227	−0.0013 −0.0006 −0.0008 −0.0008
**1b**	I1···O1A I1···O2C I1B···O2A I1B···O1C	2.628 2.664 2.647 2.669	−0.0345 −0.0318 −0.0330 −0.0321	0.0994 0.0943 0.0967 0.0926	−0.0255 −0.0232 −0.0243 −0.0230	0.0243 0.0227 0.0234 0.0224	−0.0012 −0.0005 −0.0008 −0.0006
**2a**	I1···O1A I1···O2A	2.479 2.996	−0.0483 −0.0141	0.1163 0.0557	−0.0379 −0.0092	0.0314 0.0114	−0.0064 0.0023
**2b**	I1···O1A I1···O2A	2.480 2.961	−0.0483 −0.0151	0.1151 0.0597	−0.0377 −0.0101	0.0312 0.0124	−0.0065 0.0023
**3a**	I1···O1A I1···O5A I1B···O3A I1B···O5A I1B···O6A I1C···O4A I1C···O1S	2.479 2.957 2.692 2.833 3.037 2.630 2.828	−0.0477 −0.0171 −0.0285 −0.0240 −0.0171 −0.0358 −0.0250	0.1186 0.0590 0.0939 0.0702 0.0554 0.0973 0.0736	−0.0378 −0.0107 −0.0211 −0.0155 −0.0109 −0.0261 −0.0165	0.0318 0.0126 0.0217 0.0162 0.0123 0.0243 0.0171	−0.0061 0.0019 0.0006 0.0007 0.0014 −0.0018 0.0007
**3b**	I1···O1A I1···O5A I1B···O3A I1B···O5A I1B···O6A I1C···O4A I1C···O1S	2.474 2.956 2.697 2.766 3.097 2.640 2.771	−0.0482 −0.0170 −0.0283 −0.0274 −0.0154 −0.0353 −0.0272	0.1195 0.0592 0.0935 0.0784 0.0512 0.0957 0.0810	−0.0383 −0.0108 −0.0210 −0.0183 −0.0097 −0.0254 −0.0185	0.0321 0.0126 0.0216 0.0185 0.0112 0.0238 0.0189	−0.0062 0.0019 0.0007 0.0002 0.0015 −0.0016 0.0005

## Data Availability

Data are available on request from the authors.

## Supplementary Materials

The supporting information can be downloaded at: https://www.mdpi.com/article/10.3390/ijms241914642/s1.
