# Supplementary material for: Halogen Bond-Involving Self-Assembly of Iodonium Carboxylates: Adding a Dimension to Supramolecular Architecture
Source: Int J Mol Sci. 2023 Sep 27;24(19):14642. doi: 10.3390/ijms241914642 (PMC10573078; doi:10.3390/ijms241914642)
Supplement: Supplementary file 1 [file ijms-24-14642-s001.zip › ijms-2611112-supplementary.pdf]

SUPPORTING INFORMATION

Halogen Bond-Involving Self-assembly of Iodonium Carboxylates: Adding a Dimension to Supramolecular Architecture

Amirbek D. Radzhabov,[1] Alyona I. Ledneva,[1] Natalia S. Soldatova,[1] Irina I. Fedorova,[2,3] Daniil M. Ivanov,[1,2] Alexey A. Ivanov,[1] Mekhman S. Yusubov,[1] Vadim Yu. Kukushkin,[2,4] Pavel S. Postnikov[1,5]*

1 Research School of Chemistry and Applied Biomedical Sciences, Tomsk Polytechnic University, Tomsk 634034, Russian Federation
2 Institute of Chemistry, Saint Petersburg State University, Saint Petersburg 199034, Russian Federation
3 Department of Mathematics and Mechanics, Saint Petersburg State University, Saint Petersburg 199034, Russian Federation
4 Institute of Chemistry and Pharmaceutical Technologies, Altai State University, 656049 Barnaul, Russia;
5 Department of Solid State Engineering, Institute of Chemical Technology, Prague 16628, Czech Republic

* Correspondence: postnikov@tpu.ru

Table of contents

Table S1. Geometrical parameters of C–X···Y (X = Cl, Br) XBs in the structures of **1**–**3**. S2

Table S2. Parameters in (3, –1) bond critical points (the electron density with sign of λ_2 sign(λ_2)ρ(r) in *e*/bohr^3, Laplacian of electron density $\nabla^2\rho$(r) in *e*/bohr^5, the local electronic energy density H_b, local electronic potential energy density *V*(r), local electronic kinetic energy density *G*(r) in hartrees/bohr^3) corresponding to the Cl···Cl (Br···Br), Cl···C (Br···C), Cl···O (Br···O) XBs and HBs in crystal models of all structures. S2

Figure S1. XB C–X···Y in **1a** (A) and **1b** (B). S3

Figure S2. XB C–X···Y in **3a** (A, C) and **3b** (B, D). S3

Table S3. Crystal structure refinement. S4

Crystal growth S5

Synthetic procedures S5

NMR spectra of **1a**,**b** and **3a**,**b** S8

Processing of Cambridge Structure Database S16

References S17

Table S1. Geometrical parameters of C–X···Y (X = Cl, Br) XBs in the structures of **1**–**3**.

**Cation type**[a]	**XB**	***d*(C–X···Y)**	**∠(C–X···Y)**	**Nc**[b]	***d*(C–X···Y)**	**∠(C–X···Y)**	**Nc**[b]
		**1a**			**1b**		
A	C4–X1···X2B	3.5223(17)	171.99(14)	1.01	3.5522(12)	173.00(19)	0.96
B	C10B–X2B···X1B	3.5513(17)	163.54(16)	1.01	3.5578(12)	168.7(2)	0.96
		**3a**			**3b**		
A	C4–X1···X2	3.539(3)	176.6(3)	1.01	3.5969(11)	175.8(2)	0.97
B	C4B–X1B···O1A	3.090(6)	154.6(3)	0.94	3.078(5)	155.4(2)	0.91
C	-	-	-	-	-	-	-

Table S2. Parameters in (3, –1) bond critical points (the electron density with sign of λ_2 sign(λ_2)ρ(r) in *e*/bohr^3, Laplacian of electron density $\nabla^2\rho$(r) in *e*/bohr^5, the local electronic energy density H_b, local electronic potential energy density *V*(r), local electronic kinetic energy density *G*(r) in hartrees/bohr^3) corresponding to the Cl···Cl (Br···Br), Cl···C (Br···C), Cl···O (Br···O) XBs and HBs in crystal models of all structures.

**Structure**	**Contact**	***l***	**sign(λ_2)ρ(r)**	**$\nabla^2\rho$(r)**	***V*(r)**	***G*(r)**	**H_b**
**1a**	Cl1···Cl2B	3.522	–0.0058	0.0203	–0.0026	0.0038	0.0012
	Cl2B···Cl1B	3.551	–0.0056	0.0192	–0.0025	0.0036	0.0012
**1b**	Br1···Br2B	3.552	–0.0080	0.0275	–0.0038	0.0054	0.0015
	Br2B···Br1B	3.558	–0.0080	0.0273	–0.0038	0.0053	0.0015
**2a**	Cl1···C11	3.182	–0.0080	0.0295	–0.0040	0.0057	0.0017
	Cl2···C5	3.355	–0.0067	0.0235	–0.0032	0.0045	0.0014
**2b**	Br1···C11	3.249	–0.0085	0.0295	–0.0043	0.0058	0.0015
	Br2···C5	3.392	–0.0072	0.0251	–0.0036	0.0049	0.0013
**3a**	Cl1···Cl2	3.539	–0.0057	0.0198	–0.0026	0.0038	0.0012
	Cl1B···O1A	3.090	–0.0088	0.0365	–0.0049	0.0070	0.0021
	H1SA···O2A	1.769	–0.0378	0.1180	–0.0325	0.0310	–0.0015
	H1SB···O2S	1.781	–0.0368	0.1118	–0.0309	0.0294	–0.0015
	H2SB···O6A	1.767	–0.0369	0.1232	–0.0327	0.0317	–0.0009
	H2SA···O3A	1.930	–0.0259	0.0885	–0.0201	0.0211	0.0010
**3b**	Br1···Br2	3.597	–0.0074	0.0255	–0.0035	0.0049	0.0014
	Br1B···O1A	3.078	–0.0108	0.0435	–0.0067	0.0088	0.0021
	H1SA···O2A	1.737	–0.0404	0.1217	–0.0353	0.0328	–0.0024
	H1SB···O2S	1.824	–0.0339	0.1032	–0.0275	0.0266	–0.0008
	H2SA···O6A	1.713	–0.0426	0.1296	–0.0385	0.0354	–0.0030
	H2SB···O3A	1.869	–0.0288	0.0956	–0.0229	0.0234	0.0005

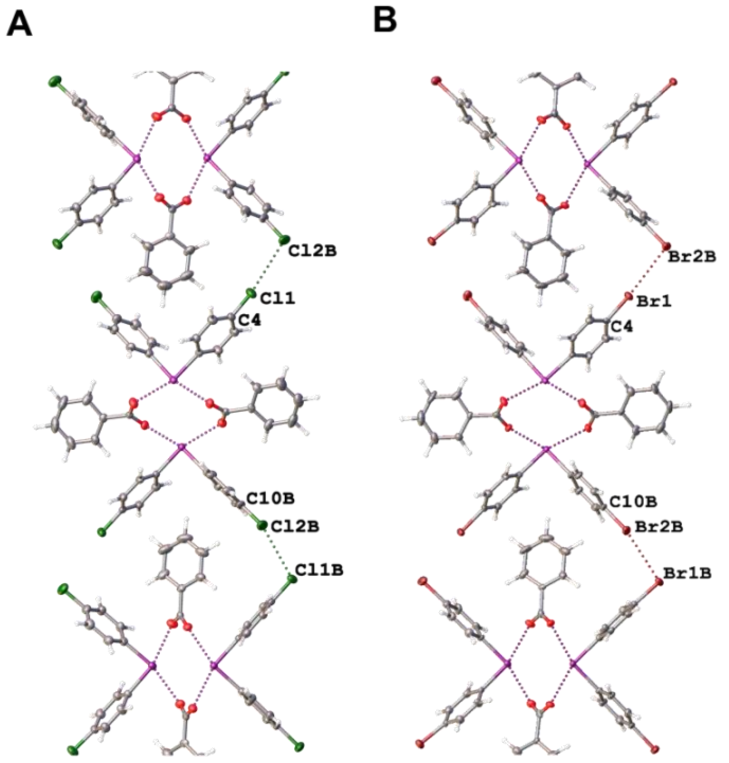

Figure S1. XB C–X···Y in **1a** (A) and **1b** (B).

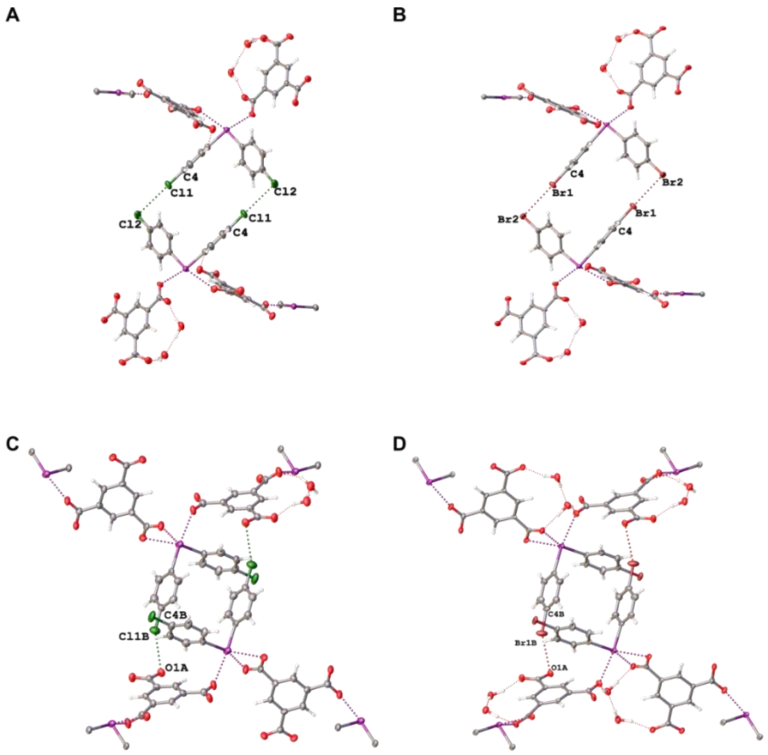

Figure S2. XB C–X···Y in **3a** (A, C) and **3b** (B, D).

Table S3. Crystal structure refinement.

Identification code	**1a**	**1b**	**2a**	**2b**	**3a**	**3b**
CCDC number	2291471	2291472	2291473	2291475	2291476	2291477
Empirical formula	$C_{19}H_{13}Cl_2IO_2$	$C_{19}H_{13}Br_2IO_2$	$C_{32}H_{20}Cl_4I_2O_4$	$C_{32}H_{20}Br_4I_2O_4$	$C_{45}H_{31}Cl_6I_3O_8$	$C_{45}H_{31}Br_6I_3O_8$
Formula weight	471.11	1120.03	864.08	1041.92	1293.10	1559.86
Temperature, K	100(2)	100(2)	100(2)	100(2)	100(2)	100(2)
Crystal system	orthorhombic	orthorhombic	triclinic	triclinic	monoclinic	monoclinic
Space group	Pbca	Pbca	P-1	P-1	$P2_1/n$	$P2_1/n$
a, Å	19.8620(6)	20.2173(9)	7.3451(4)	7.5887(5)	11.7190(2)	11.8708(2)
b, Å	9.2439(2)	9.1860(5)	9.6604(5)	9.5201(7)	28.4879(6)	28.5948(4)
c, Å	40.3145(11)	40.790(2)	10.9579(5)	11.0650(7)	14.0194(3)	14.2162(2)
α, °	90	90	104.536(4)	77.605(6)	90	90
β, °	90	90	90.659(4)	88.895(5)	96.078(2)	95.2910(10)
γ, °	90	90	99.660(5)	80.537(6)	90	90
Volume, Å^3	7401.90(3)	7575.3(7)	740.80(7)	770.03(9)	4654.06(16)	4805.03(13)
Z	8	8	1	1	4	4
ρ_{calc}, g/cm^3	1.691	1.964	1.937	2.247	1.845	2.156
μ, mm^{-1}	2.027	5.918	2.522	7.269	19.427	21.594
F(000)	3680.0	4256.0	418.0	490.0	2504.0	2936.0
Crystal size, mm^3	0.7 × 0.3 × 0.08	1.5 × 1 × 0.5	0.18 × 0.12 × 0.08	0.6 × 0.5 × 0.5	0.18 × 0.15 × 0.13	0.2 × 0.18 × 0.17
Radiation	Mo Kα (λ = 0.71073)	Mo Kα (λ = 0.71073)	Mo Kα (λ = 0.71073)	Mo Kα (λ = 0.71073)	Cu Kα (λ = 1.54184)	Cu Kα (λ = 1.54184)
2Θ range for data collection, °	4.532 to 57.538	4.474 to 57.158	5.064 to 58.676	4.44 to 57.27	6.206 to 134.996	6.182 to 129.974
Index ranges	$-20 \le h \le 26$, $-12 \le k \le 12$, $-52 \le l \le 50$	$-16 \le h \le 25$, $-10 \le k \le 11$, $-47 \le l \le 54$	$-8 \le h \le 9$, $-11 \le k \le 12$, $-14 \le l \le 14$	$-10 \le h \le 9$, $-12 \le k \le 12$, $-14 \le l \le 14$	$-14 \le h \le 13$, $-34 \le k \le 33$, $-16 \le l \le 15$	$-12 \le h \le 13$, $-33 \le k \le 33$, $-16 \le l \le 14$
Reflections collected	45164	36807	6076	9155	51478	41302
Independent reflections	8636 [R_{int} = 0.0539, R_{sigma} = 0.0517]	8713 [R_{int} = 0.0869, R_{sigma} = 0.0995]	3384 [R_{int} = 0.0239, R_{sigma} = 0.0447]	3433 [R_{int} = 0.0285, R_{sigma} = 0.0342]	8339 [R_{int} = 0.1651, R_{sigma} = 0.0809]	8172 [R_{int} = 0.0946, R_{sigma} = 0.0508]
Data/restraints/parameters	8636/0/433	8713/0/427	3384/0/190	3433/0/190	8339/2/565	8172/2/565
Goodness-of-fit on F^2	1.037	1.027	1.046	1.057	1.060	1.027
Final R indexes [I≥2σ (I)]	R_1 = 0.0428, wR_2 = 0.0830	R_1 = 0.0628, wR_2 = 0.0998	R_1 = 0.0274, wR_2 = 0.0558	R_1 = 0.0254, wR_2 = 0.0510	R_1 = 0.0597, wR_2 = 0.1565	R_1 = 0.0461, wR_2 = 0.1171
Final R indexes [all data]	R_1 = 0.0680, wR_2 = 0.0910	R_1 = 0.1098, wR_2 = 0.1121	R_1 = 0.0320, wR_2 = 0.0582	R_1 = 0.0318, wR_2 = 0.0526	R_1 = 0.0735, wR_2 = 0.1673	R_1 = 0.0500, wR_2 = 0.1200
Largest diff. peak/hole, e·Å^{-3}	0.90/-0.62	0.94/-1.23	1.35/-0.68	1.19/-0.91	2.16/-1.97	2.84/-1.24

Crystal growth

Diaryliodonium benzoates **1**.

Crystals of **1** were grown by slow evaporation of methanol solution of **1** at ambient temperature.

Diaryliodonium terephtalates **2**.

Crystals of **2** were grown by slow diffusion of tetrabutylammonium terephthalate methanol solution to diaryliodonium trifluoromethanesulfonate methanol solution in ampule at ambient temperature. Solution of diaryliodonium salt was carefully added, avoiding significant mixing.

Diaryliodonium trimesates **3**.

Crystals of **3** suitable for X-Ray diffraction were grown by slow diffusion of tetrabutylammonium terephthalate solution in water/acetonitrile mixture (1:1) to diaryliodonium trifluoromethanesulfonate in acetonitrile at ambient temperature. Solution of diaryliodonium salt was carefully added, avoiding significant mixing.

Synthetic procedures

Synthesis of diaryliodonium benzoates 1. A solution of a diaryliodonium trifluoromethanesulfonate [1] (1 mmol) in methanol/water mixture (1 mL) was added dropwise to solution of potassium benzoate (3 mmol, 481 mg) in water (5 mL) at RT, the reaction mixture was stirred for 30 min and the precipitate formed was filtered off and washed with water (3×5 mL). The obtained diaryliodonium benzoates 1 were dried under reduced pressure.

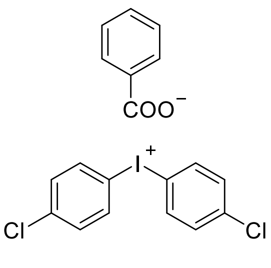

bis(4-chlorophenyl)iodonium benzoate (1a). The reaction of bis(4-chlorophenyl)iodonium trifluoromethanesulfonate (1 mmol, 499 mg) according to described procedure afforded 457 mg (97%) of **1a** isolated as the colorless crystalline solid; mp 152–153 °C. ^{1}H NMR (400 MHz, DMSO-d_6) δ 8.18 (d, J = 8.8 Hz, 4H), 7.75 (d, J = 6.8 Hz, 2H), 7.52 (d, J = 8.4 Hz, 4H), 7.32–7.23 (m, 3H). ^{13}C{^{1}H} (100 MHz, DMSO-d_6) δ 169.7, 138.8, 136.8, 136.2, 131.1, 129.2, 129.0, 127.3, 118.9. HRMS (ESI+) *m/z* calcd. for $[M]^+$ $C_{12}H_8Cl_2I^+$: 348.9042, found 348.9045.

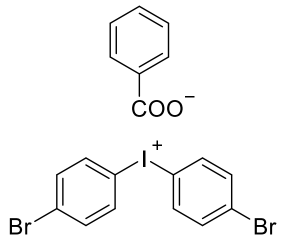

bis(4-bromophenyl)iodonium benzoate (1b). The reaction of bis(4-bromophenyl)iodonium trifluoromethanesulfonate (1 mmol, 588 mg) according to described procedure afforded 527 mg (94%) of **1b** isolated as the colorless crystalline solid; mp 158–159 °C. ^{1}H NMR (400 MHz, DMSO-d_6) δ 8.09 (d, J = 8.0 Hz, 4H), 7.79 (d, J = 6.8 Hz, 2H), 7.65 (d, J = 8.4 Hz, 4H), 7.37–7.28 (m, 3H). ^{13}C{^{1}H} (100 MHz, DMSO-d_6) δ 169.3, 136.9, 134.0, 129.9, 129.8, 129.0, 127.7, 125.1, 119.4. HRMS (ESI+) *m/z* calcd. for $[M]^+$ $C_{12}H_8Br_2I^+$: 436.8032, found 436.8039.

Synthesis of diaryliodonium terephtalates 2. To a solution of a diaryliodonium trifluoromethanesulfonate [1] (2.2 mmol) in methanol/water mixture (5 mL, 1:1) the solution of tetrabutylammonium terephthalate (1 mmol, 649 mg) in methanol (1 mL) was added dropwise at RT. The reaction mixture was stirred for 30 min and the precipitate formed was filtered off and washed with water (3×5 mL). The obtained diaryliodonium terephthalates **2** were dried under reduced pressure.

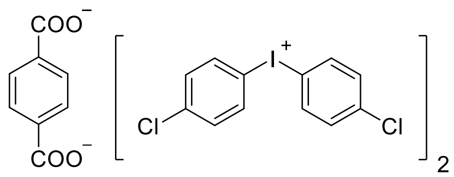

bis(4-chlorophenyl)iodonium terephthalate (2a). The reaction of bis(4-chlorophenyl)iodonium trifluoromethanesulfonate (2.2 mmol, 1.1 g) according to described procedure afforded 682 mg (79%) of **2a** isolated as the colorless crystalline solid; mp 200–202 °C. Elemental analysis (Found; C 43.43%, H 2.49%, Calcd. for $C_{32}H_{20}Cl_4I_2O_4$: C 44.48%, H 2.33%). HRMS (ESI+) *m/z* calcd. for $[M]^+$ $C_{12}H_8Cl_2I^+$: 348.9042, found 348.9042.

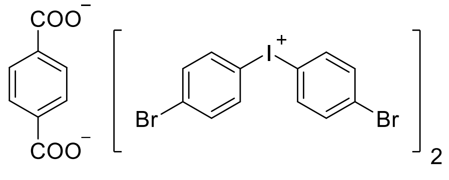

bis(4-bromophenyl)iodonium terephthalate (2b). The reaction of bis(4-bromophenyl)iodonium trifluoromethanesulfonate (2.2 mmol, 1.29 g) according to described procedure afforded 885 mg (85%) of **2b** isolated as the colorless crystalline solid; mp

191–193 °C. Elemental analysis (Found; C 36.82%, H 2.24%, Calcd. for $C_{32}H_{20}Br_4I_2O_4$: C 36.89%, H 1.93%). HRMS (ESI+) *m/z* calcd. for $[M]^+$ $C_{12}H_8Br_2I^+$: 436.8032, found 436.8043.

Synthesis of diaryliodonium trimesates 3. To a solution of tetrabutylammonium trimesate (1 mmol, 934 mg) in water/acetonitrile mixture (5 mL, 1:1) a solution of diaryliodonium trifluoromethanesulfonate[1] (3.3 mmol) in acetonitrile (5 mL) was added dropwise at RT. The reaction mixture was stirred for 30 min and the precipitate formed was filtered off and washed with water (3×5 mL). The product **3** was dried in the air.

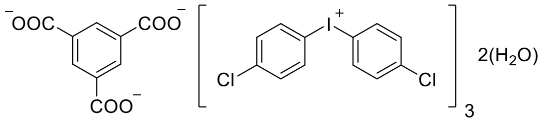

bis(4-chlorophenyl)iodonium benzene-1,3,5-tricarboxylate dihydrate (3a). The reaction of bis(4-chlorophenyl)iodonium trifluoromethanesulfonate (3.3 mmol, 1.65 g) according to described procedure afforded 905 mg (72%) of **3a** isolated as the colorless crystalline solid; mp 152–154 °C. ^{1}H NMR (400 MHz, DMSO-d_6) δ 8.23–8.10 (m, 15H), 7.49 (d, *J* = 8.4 Hz, 12H). ^{13}C{^{1}H} (100 MHz, DMSO-d_6) δ 170.5, 136.7, 136.6, 135.9, 131.2, 130.9, 119.8. Elemental analysis (Found; C 42.18%, H 2.56%, Calcd. for $C_{45}H_{31}Cl_6I_3O_8$: C 41.80%, H 2.42%). HRMS (ESI+) *m/z* calcd. for $[M]^+$ $C_{12}H_8Cl_2I^+$: 348.9042, found 348.9032.

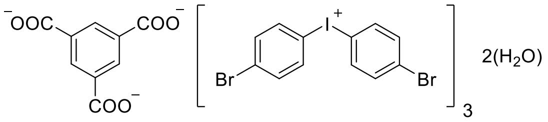

bis(4-bromophenyl)iodonium benzene-1,3,5-tricarboxylate dihydrate (3b). The reaction of bis(4-bromophenyl)iodonium trifluoromethanesulfonate (3.3 mmol, 1.94 g) according to described procedure afforded 1.26 g (83%) of **3b** isolated as the colorless crystalline solid; mp 146–148 °C. ^{1}H NMR (400 MHz, DMSO-d_6) δ 8.17 (s, 3H), 8.07 (d, *J* = 8.4 Hz, 12H), 7.62 (d, *J* = 8.4 Hz, 12H). ^{13}C{^{1}H} (100 MHz, DMSO-d_6) δ 170.5, 136.8, 136.6, 133.8, 131.2, 124.7, 120.5. Elemental analysis (Found; C 34.73%, H 2.00%, Calcd. for $C_{45}H_{31}Br_6I_3O_8$: C 34.65%, H 2.00%). HRMS (ESI+) *m/z* calcd. for $[M]^+$ $C_{12}H_8Br_2I^+$: 436.8032, found 436.8029.

NMR spectra of 1a,b and **3a,b**

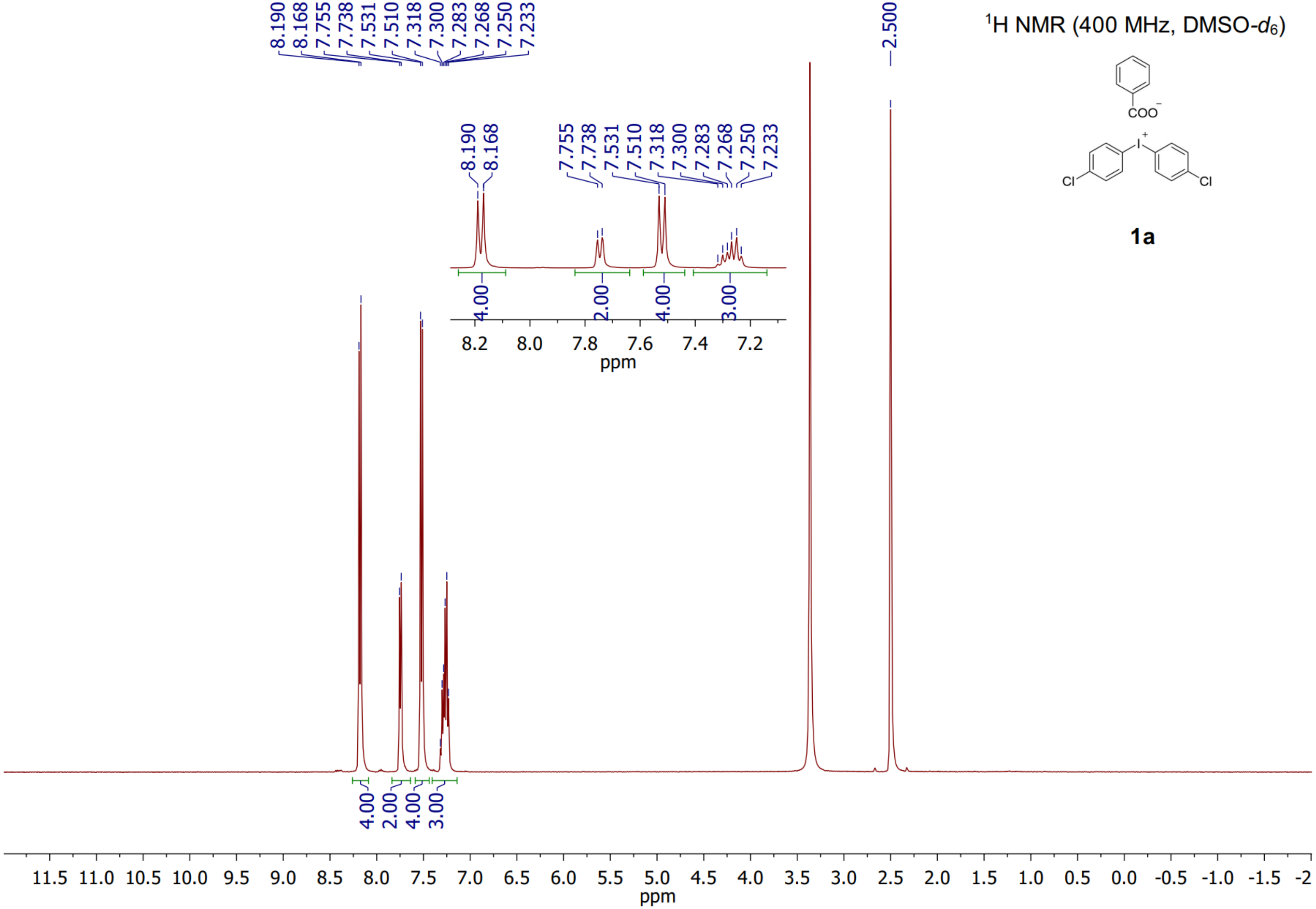

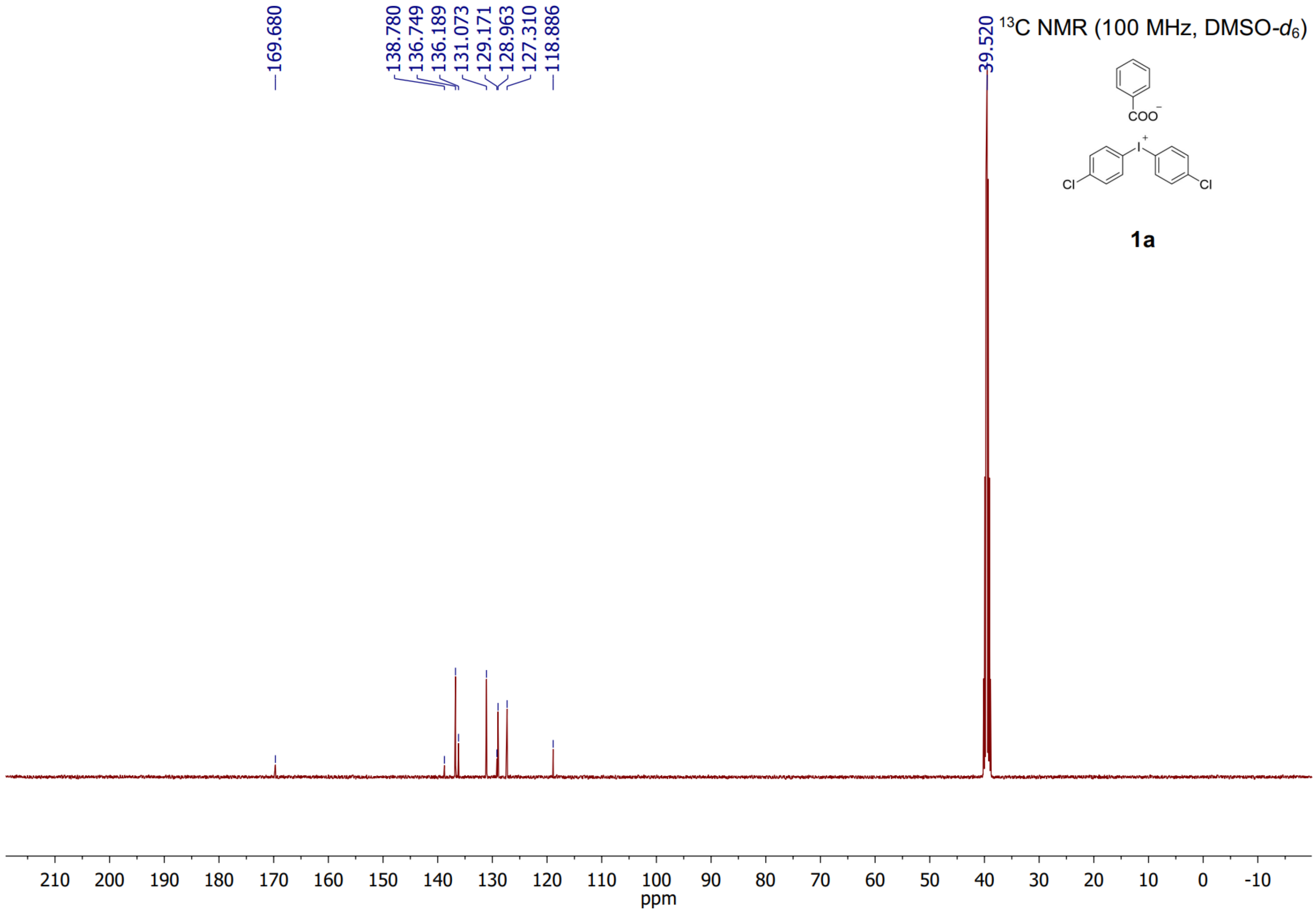
169.680
138.780
136.749
136.189
131.073
129.171
128.963
127.310
118.886
39.520
13C NMR (100 MHz, DMSO-d6)
COO−
I+
Cl
Cl
1a
210 200 190 180 170 160 150 140 130 120 110 100 90 80 70 60 50 40 30 20 10 0 -10
ppm

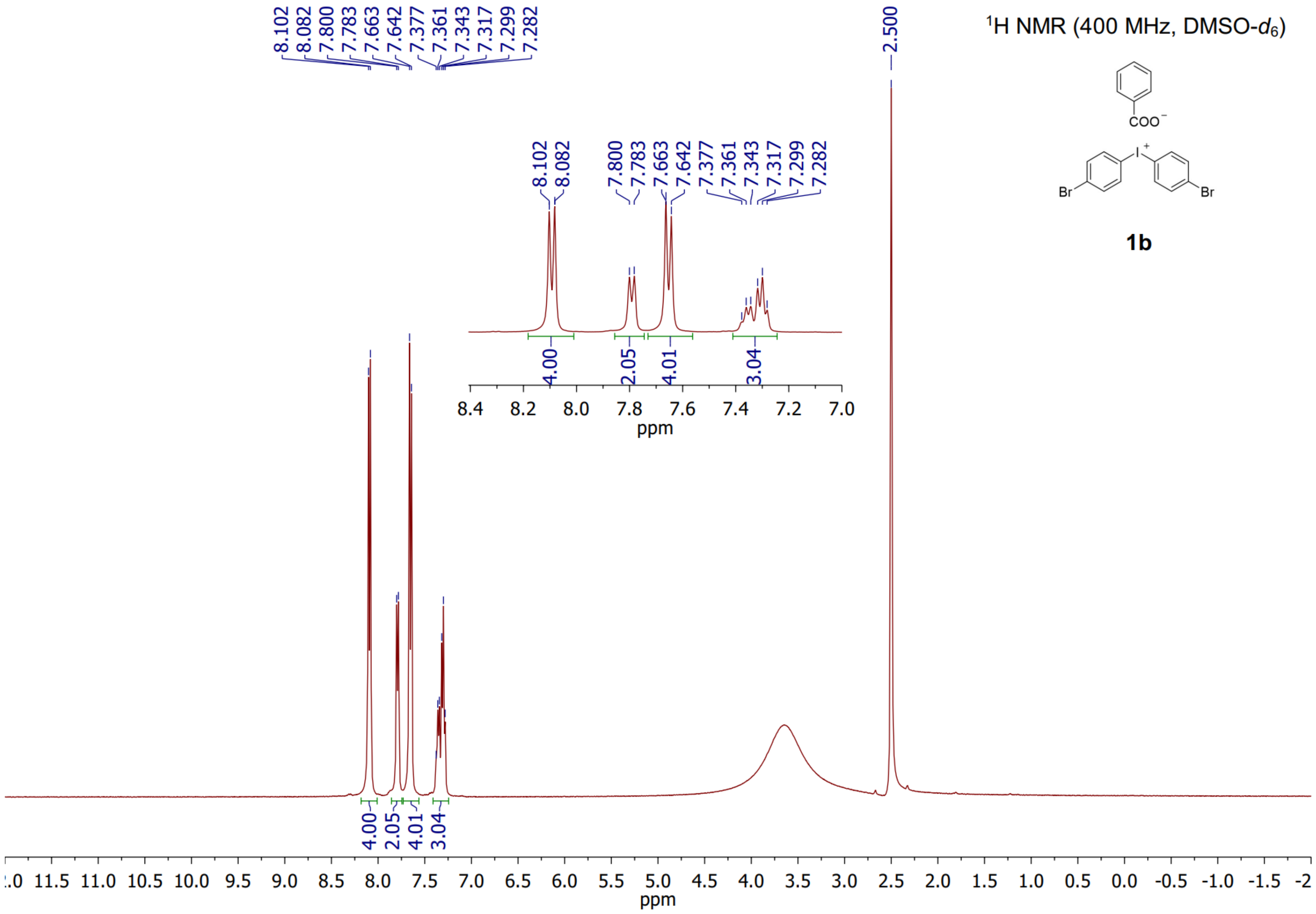

1H NMR (400 MHz, DMSO-d6)
8.102
8.082
7.800
7.783
7.663
7.642
7.377
7.361
7.343
7.317
7.299
7.282
2.500
COO−
I+
Br
Br
4.00
2.05
4.01
3.04
8.4 8.2 8.0 7.8 7.6 7.4 7.2 7.0
ppm
11.5 11.0 10.5 10.0 9.5 9.0 8.5 8.0 7.5 7.0 6.5 6.0 5.5 5.0 4.5 4.0 3.5 3.0 2.5 2.0 1.5 1.0 0.5 0.0 -0.5 -1.0 -1.5
ppm

1b

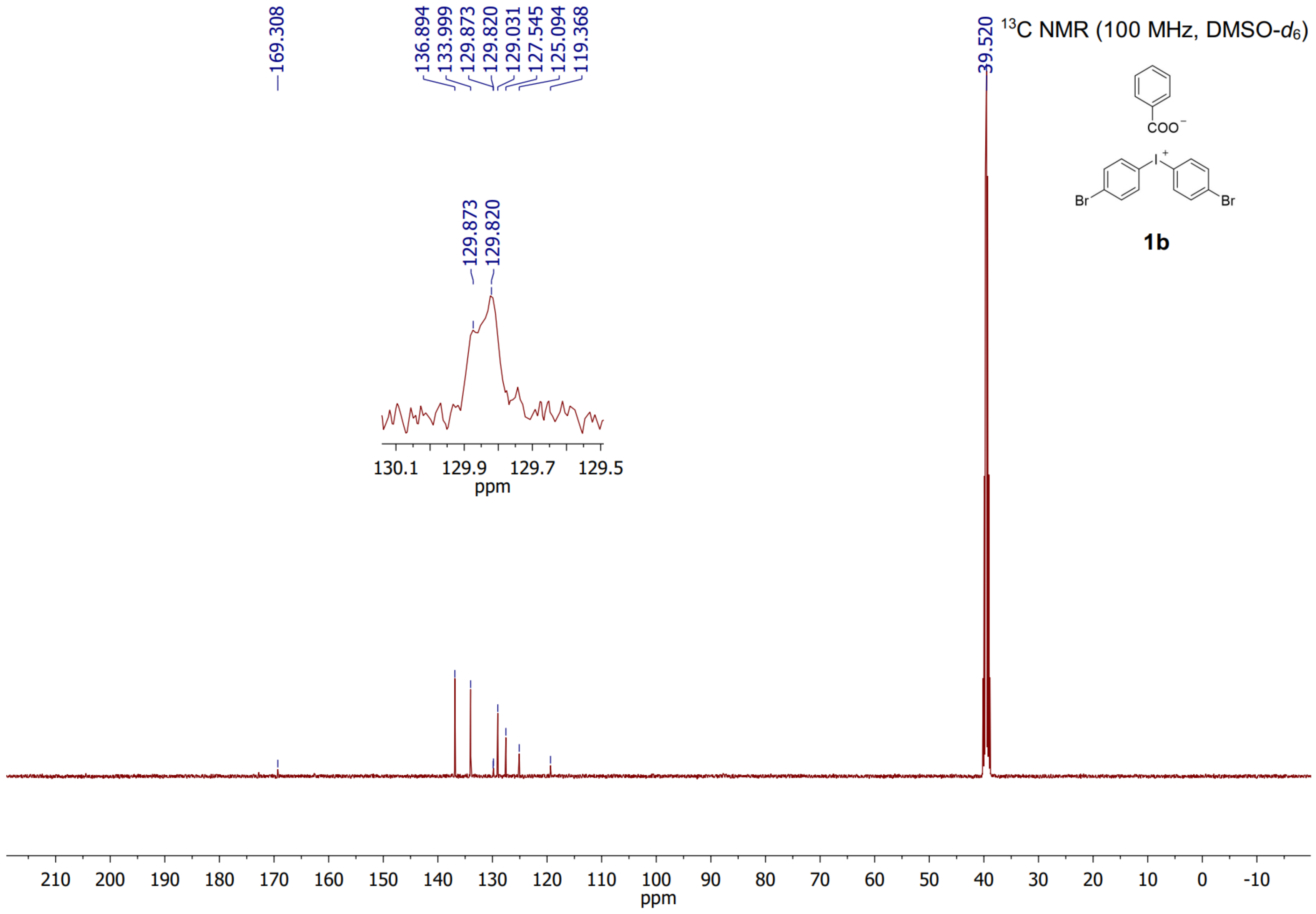

169.308
136.894
133.999
129.873
129.820
129.031
127.545
125.094
119.368
39.520
13C NMR (100 MHz, DMSO-d6)
COO−
I+
Br
Br
1b
129.873
129.820
130.1
129.9
129.7
129.5
ppm
210
200
190
180
170
160
150
140
130
120
110
100
90
80
70
60
50
40
30
20
10
0
-10
ppm

^{1}H NMR (400 MHz, DMSO-d_6)

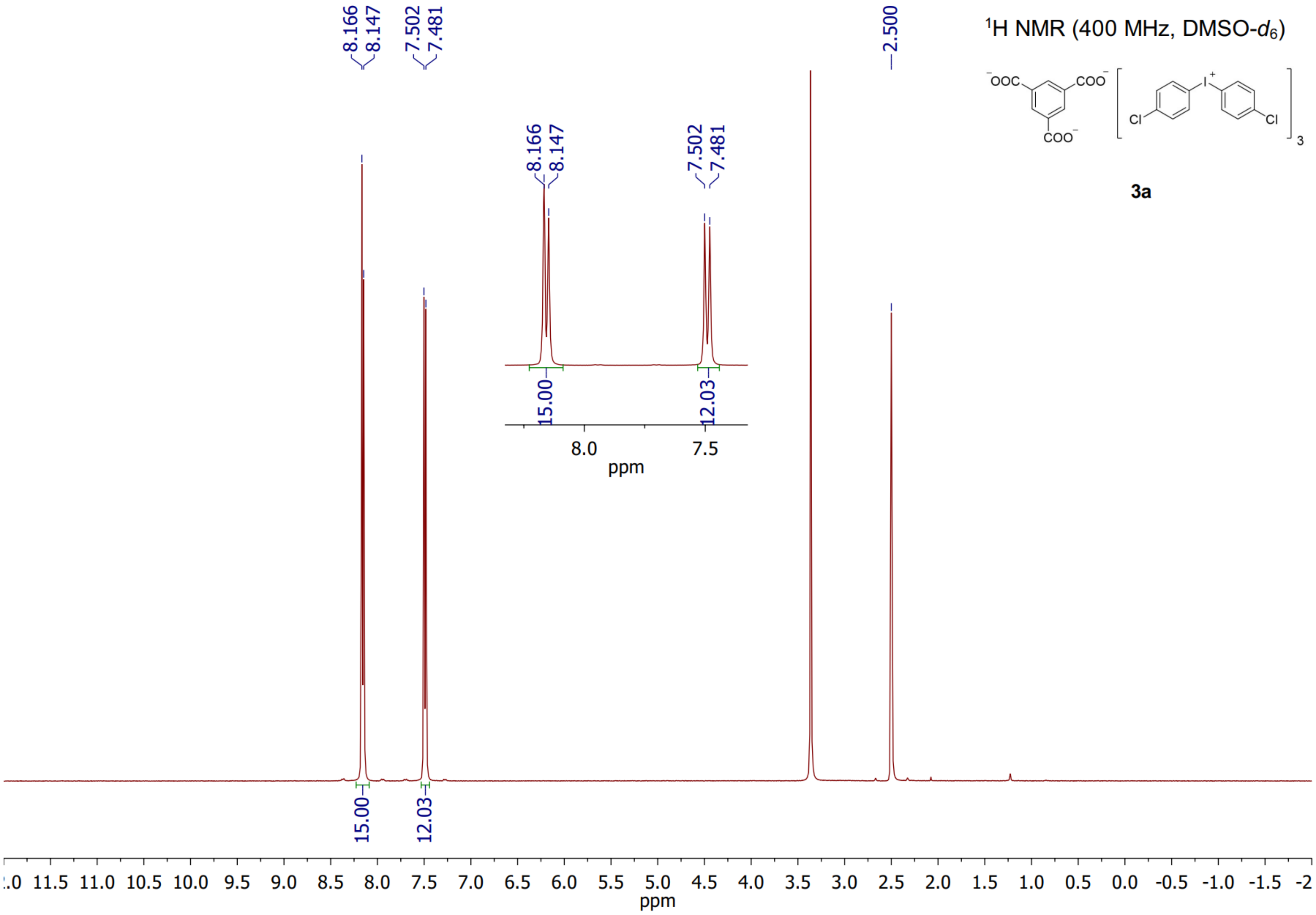

3a

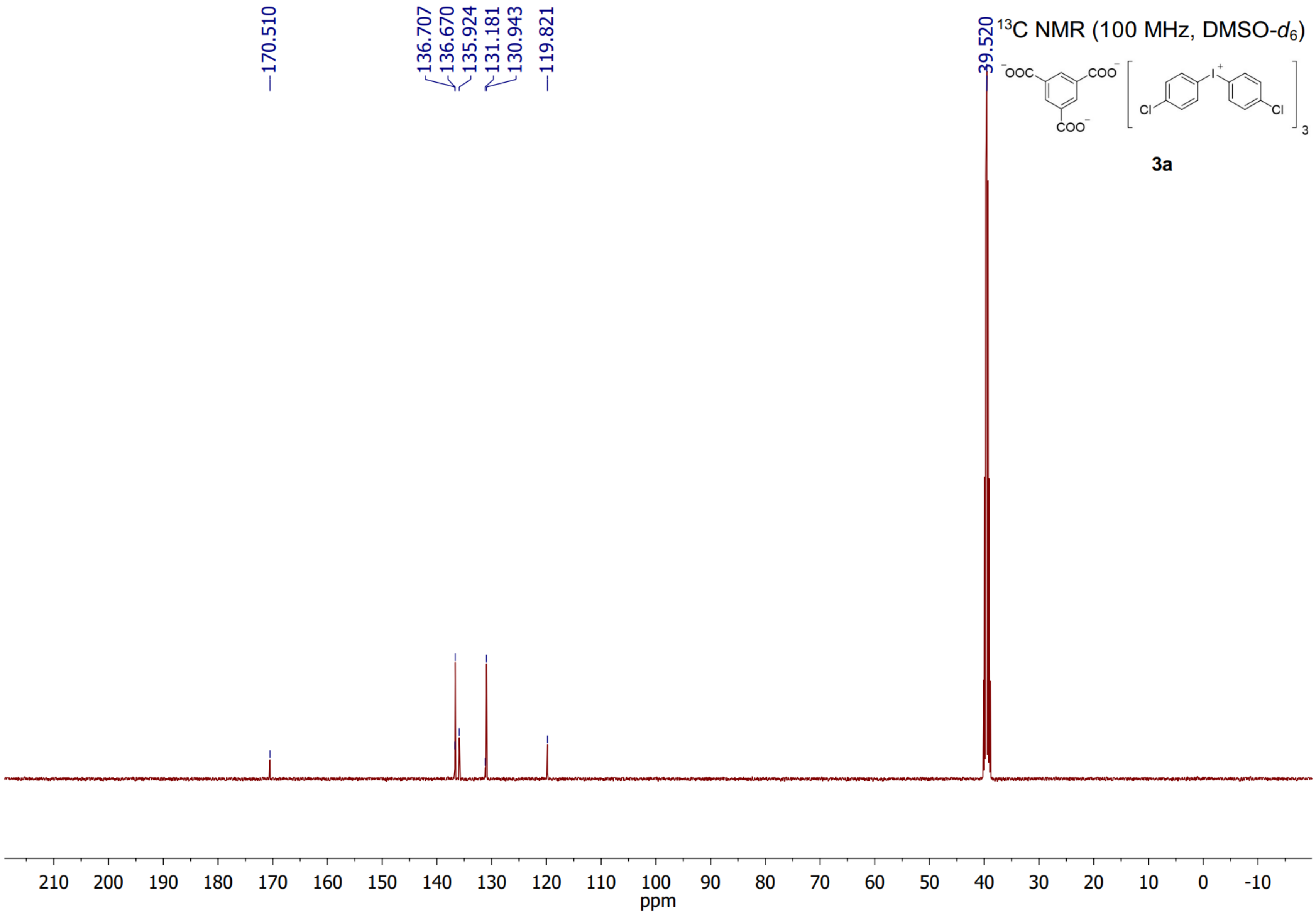

170.510
136.707
136.670
135.924
131.181
130.943
119.821
39.520
^{13}C NMR (100 MHz, DMSO-d_6)
3a
210 200 190 180 170 160 150 140 130 120 110 100 90 80 70 60 50 40 30 20 10 0 -10
ppm

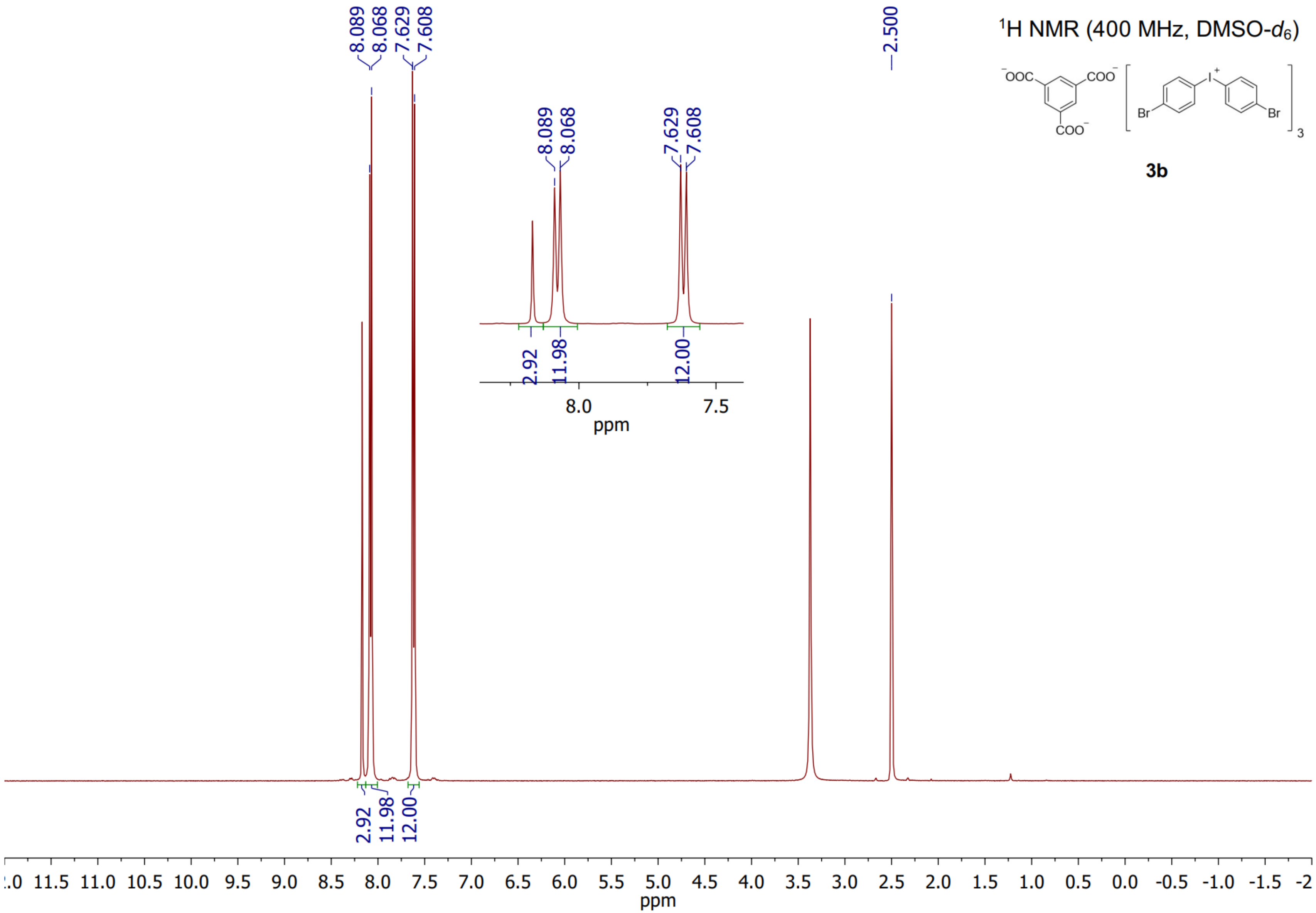

^{1}H NMR (400 MHz, DMSO-d_6)
3b
8.089
8.068
7.629
7.608
2.500
2.92
11.98
12.00
ppm

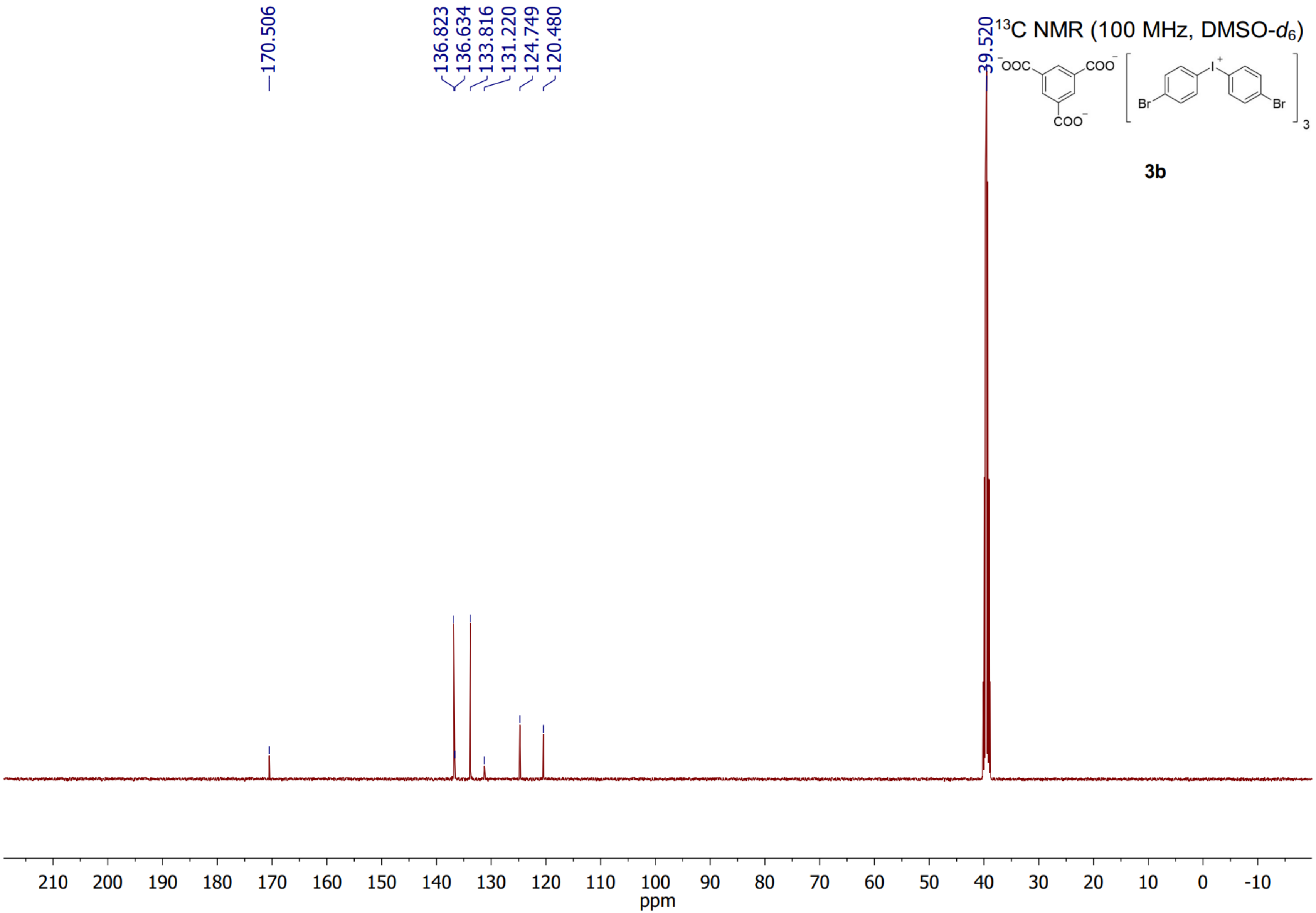

170.506
136.823
136.634
133.816
131.220
124.749
120.480
39.520
13C NMR (100 MHz, DMSO-d6)
210 200 190 180 170 160 150 140 130 120 110 100 90 80 70 60 50 40 30 20 10 0 -10
ppm
−OOC
COO−
COO−
Br
I+
Br
3

3b

Processing of Cambridge Structure Database

Processing of the Cambridge Structure Database (v 5.44) was performed using the ConQuest module (v 2023.2). To find accessible iodonium species, we analyzed 408 structures obtained by single-crystal XRD, including the C–I^+–C motif. The type of the supramolecular assembly was determined manually for each structure using Olex2-1.5 software.

References

1. Bielawski, M.; Zhu, M.; Olofsson, B. Efficient and General One-Pot Synthesis of Diaryliodonium Triflates: Optimization, Scope and Limitations. *Adv. Synth. Catal.* **2007**, *349*, 2610–2618, doi:10.1002/adsc.200700373.
